# Heavy Metal Uptake by Herbs. V. Metal Accumulation and Physiological Effects Induced by Thiuram in *Ocimum basilicum* L.

**DOI:** 10.1007/s11270-017-3508-0

**Published:** 2017-08-17

**Authors:** Dorota Adamczyk-Szabela, Zdzisława Romanowska-Duda, Katarzyna Lisowska, Wojciech M. Wolf

**Affiliations:** 1 Lodz University of Technology, Institute of General and Ecological Chemistry, Zeromskiego 116, 90-924 Lodz, Poland; 2 University of Lodz, Laboratory of Plants Ecophysiology, Faculty of Biology and Environmental Protection, Banacha 12/16, 90-237 Lodz, Poland

**Keywords:** Thiuram, Basil, Heavy metal bioaccumulation and translocation, Fungicide persistence, Farmland soils

## Abstract

Basil (*Ocimum basilicum* L.) is extensively cultivated as either an important spice and food additive or a source of essential oil crucial for the production of natural phenylpropanoids and terpenoids. It is frequently attacked by fungal diseases. The aim of the study was to estimate the impact of thiuram contact time on the uptake of manganese, cobalt, nickel, copper, zinc, cadmium, and lead by *Ocimum basilicum* L*.* The relevant plant physiological parameters were also investigated. Two farmland soils typical for the Polish rural environment were used. Studies involved soil analyses, bioavailable, and total forms for all investigated metals, chlorophyll content, and gas exchange. Atomic absorption spectrometry was used to determine concentration of all elements. Analysis of variance proved hypothesis that thiuram treatment of basil significantly influences metal transfer from soil and their concentration in roots and aboveground parts. This effect is mostly visible on the 14th day after the fungicide administration. Thiuram modifies mycoflora in the rhizosphere zone and subsequently affects either metal uptake from the soil environment or their further migration within the basil plant. Notable, those changes are more evident for basil planted in mineral soil as compared to organic soil with higher buffering capacity.

**Electronic supplementary material:**

The online version of this article (doi:10.1007/s11270-017-3508-0) contains supplementary material, which is available to authorized users.

## Introduction

Medical plants are extensively used and consumed all over the word (Basgel and Erdemoglu 2006). *Ocimum basilicum* L. (sweet basil) is an annual, green-leaved herb (*Lamiaceae* family) that has been widely used as a medicinal and seasoning plant for centuries (Stefan et al. 2013; Beatovic et al. 2015). Basil is extensively cultivated as either an important spice and food additive or a source of essential oil crucial for the production of natural phenylpropanoids and terpenoids (Bazaid et al. 2013). It demonstrates wide antibacterial (Koba et al. 2009; Carovic-Stanko et al. 2010; Moghaddam et al. 2011), antifungal (Adigüzel et al. 2005), immunomodulatory (Tsai et al. 2011), antioxidant (Salles Trevisan et al. 2006; Politeo et al. 2007; Hussain et al. 2008; Paduraru et al. 2008; Sekar et al. 2009; Taie et al. 2010), cardiotonic (Muralidharan and Dhananjayan 2004), antihyperglycemic, hypolipidemic (Zeggwagh et al. 2007), and anticonvulsant (Ismail 2006) activities. Notably, composition and activity of these oils depend strongly on agricultural practices and environmental conditions (Kandil et al. 2009; Esetlili et al. 2016). Nowadays, basil is widely present in tropical and subtropical regions (Grayer et al. 1996; Javanmardi et al. 2002; Stefan et al. 2013). In Europe, the cultivation of this plants is concentrated mainly in the Mediterranean area (Golcz and Seidler-Łożykowska 2008), but it is also grown in east and central European countries, with Poland being one of the major suppliers (Nurzyńska-Wierdak 2012; Malinowska and Jankowski 2015). Modern cultivation practices reduced impact of pests on crops quite significantly indeed (Bianchi et al. 2006). On the contrary, the plant exposure to fungi is abundant and fungal-induced plagues pose the serious threat to contemporary agriculture. They are difficult to fight and usually lead to substantial harvest losses (Janisiewicz and Korsten 2002; Snelders et al. 2011; Bruni et al. 2016; Sexton and Howlett 2006). Additionally, food plants used for the drug or spice production should be free of pathogens. Basil is frequently attacked by fungal diseases (Zechini D’Aulerio et al. 1995; Hudaib et al. 2001). They strongly influence phytopathological status of particular plant (Hudaib et al. 2002; Bruni et al. 2005) and, in consequence, the quality of essential oil. Therefore, fungal diseases are becoming the major threat to basil farming in Europe and especially in Poland. Plantations can be efficiently protected by the least toxic contact fungicides which should be applied before the harvest, in the time which secures their complete degradation. In a plethora of organic contact fungicides, dithiocarbamates, and thiuram (tetramethylthiuram disulfide) especially play a vital role in a worldwide control of fungal plant diseases and are widely used to protect herbs, arable crops, vegetables, and decorative plants (Kitagawa et al. 2002). Thiuram mode of action is based on inhibition of the pyruvic dehydrogenase system at the fungal cell level. In particular, it hinders uptake of glucose and oxygen and, in consequence, the formation of carbon dioxide by the fungal spores (Dias 2012). It is well documented that thiuram is an effective ligand for diverse coordination species and has the ability to chelate metal ions in soil environment (Victoriano 2000a, 2000b; Filipe et al. 2013; Adamczyk-Szabela 2015). Nevertheless, to the best of our knowledge, thiuram influence on the heavy metal mobility and uptake by plants have not been widely investigated yet (Adamczyk 2006).

The crop productivity and yield as demonstrated by biomass production and plant growth rates are strongly related to the intensity of photosynthesis processes. On the contrary to the numerous experimental data on the relevant physiological and metabolic effects developed in plants cultivated under control of diverse contact fungicides (Garcia et al. 2003; Xiao et al. 2006; Petit et al. 2008), impact of dithiocarbamates on the photosynthesis has not been extensively examined so far (Sau-Man Po and Ho 1997; Dias et al. 2014).

In this paper, the effect of thiuram on physiological changes and uptake of copper, zinc, manganese, cobalt, nickel, cadmium, and lead by basil plants cultivated in the model pot experiments in mineral and organic soils is reported. This work follows our ongoing investigations on the impact of cultivation conditions on the heavy metal uptake by herbs (Adamczyk 2007; Adamczyk and Jankiewicz 2008).

## Materials and Methods

### Soil Analysis

Two soil types named hereafter A and B were applied. Soil A samples were collected on farmland (uncultivated for at least 2 years before the beginning of the experiment) located away from excessive traffic, according to the procedure as in PN-ISO 10381-4: 2007 at Słupia municipality (51° 51′N, 19° 58′; 20 km from Skierniewice, Łódź province, Poland) in October 2014. Soil B is commercially available universal garden soil enriched with organic matter produced by the Hollas Ltd.

All samples were subsequently dried in a well-ventilated place, sifted through a 2-mm stainless steel sieve, and finally stored in plastic bags. Soil pH was measured by the potentiometric method in 1 mol/dm^3^ potassium chloride solution (PN-ISO 10390: 1997). The well-established gravimetric method for the determination of soil organic matter by the mass loss at 550 °C was applied (Nelson and Sommers 1996; ASTM 2000; Schumacher 2002).

The bioavailable forms of metals were determined in either 1 or 0.5 mol L^−1^ solutions of hydrochloric acid extracts for soils A and B, respectively (PN-ISO 11259: 2001). The total metal content was measured in samples mineralized using the Anton Paar Multiwave 3000 closed system instrument. The mixture of concentrated HNO_3_ (6 mL) and HCl (2 mL) was applied (0.5000 g of soil). Metal concentrations were measured by the FAAS with the GBC Scientific Equipment 932 plus spectrometer. The soil properties are listed in Table 1.

**Table 1:** Results of soil A and B analysis

Analysis		Results	
		Soil A	Soil B
Soil pH		4.7	5.5
Organic matter (%)		2.4	61
Manganese (μg g^−1^)	Total forms	256 ± 15	107 ± 8
	Bioavailable forms	119 ± 7	77.8 ± 5.4
Cobalt (μg g^−1^)	Total forms	2.17 ± 0.42	4.43 ± 0.44
	Bioavailable forms	0.90 ± 0.14	2.61 ± 0.52
Nickel (μg g^−1^)	Total forms	8.02 ± 0.64	59.5 ± 1.8
	Bioavailable forms	1.13 ± 0.12	54.8 ± 1.9
Copper (μg g^−1^)	Total forms	4.37 ± 0.62	35.9 ± 2.1
	Bioavailable forms	0.93 ± 0.10	16.5 ± 1.4
Zinc (μg g^−1^)	Total forms	8.27 ± 0.91	204 ± 13
	Bioavailable forms	2.12 ± 0.28	100 ± 7
Cadmium (μg g^−1^)	Total forms	0.26 ± 0.01	0.77 ± 0.09
	Bioavailable forms	0.18 ± 0.02	0.37 ± 0.06
Lead (μg·g^−1^)	Total forms	7.38 ± 0.57	42.6 ± 3.0
	Bioavailable forms	4.72 ± 0.48	39.6 ± 1.1

*n* = 5

*p* = 0.95

*n* number of samples, *p* confidence level

### Preparation of Plant Material

Basil was cultivated under laboratory conditions by the well-established pot method (Adamczyk-Szabela et al. 2015) from April to July. Carefully weighted 500 g samples of soils A and B were placed in 48 plastic pots (24 pots per one soil type) with a diameter of 14 cm and a height of 20 cm. Seeds of *Ocimum basilicum* L. (P.H. Legutko Company, Poland) were sown in an amount of 0.1 g (approximately 100 seeds) per pot. All pots were kept in a growth chamber at controlled temperatures 23 ± 2 and 16 ± 2 °C for day and night, respectively. The relative humidity was limited to 70–75% while the photosynthetic active radiation (PAR) during the 16-h photoperiod was restricted to 400 μmol m^−2^ s^−1^. All plants were regularly watered by deionized water.

Main cultivation experiments were performed in two arrangements, each was related to one type of soil. A single batch consisted of four series of cultures, each with six pots giving, 24 samples for one arrangement altogether. The fourth series in a batch was cultivated as a reference without addition of a fungicide. Thiuram was applied (6 mg thiuram kg^−1^ of soil) to plants, 1 month after they had been sown. Herbs were harvested in periods of 14, 28, and 42 days after administration of fungicide. The aboveground parts of plants were cut while the roots were separated from soil by washing and rinsing with distilled water. The entire harvest was oven-dried at 45 °C to a constant weight, homogenized, and grounded.

### Determination of Metals in Basil

The dried roots and aboveground parts of basil plant (0.5 g sample) were subjected to microwave mineralization in concentrated HNO_3_ (6 mL) and HCl (1 mL) acid solutions using the Anton Paar Multiwave 3000 closed system instrument. Metal contents were determined by the FAAS using an air/acetylene flame and GAAS with the Scientific Equipment GBC 932 plus and GBC, SensAA spectrometers, respectively. Respective metal nitrates (Me(NO_3_)_2_, Merck) were used for calibration curve determinations. The reliability of the analytical procedures was checked using the certified reference material INCT-MPH-2, containing a mixture of selected Polish herbs (Dybczyński et al. 2004).

### Basil Plant Growth and Its Physiological Activity

Plant height was measured from the soil surface up to the highest part of the leaf. Index of chlorophyll content was evaluated using Konica Minolta SPAD-502, Japan, methodology in which the chlorophyll concentration is determined by measuring the leaf absorbance in the red and near-infrared regions. Readings were taken around the midrib of each leaf sample. Gas exchange (activity of net photosynthesis, stomatal conductance, intercellular concentration of carbon dioxide, and transpiration) were determined with the gas analyzer apparatus TPS-2 (Portable Photosynthesis System, USA) (Grzesik and Romanowska-Duda 2015; Piotrowski et al. 2016; Kalaji et al. 2012, 2016). All measurements were made in triplicate on separate plants.

### Statistical Analysis

A one-way analysis of variance (ANOVA) as implemented in the Microsoft Excel 2010 was used to test the impact of thiuram contact time on the heavy metal accumulation by basil plant cultivated in soils A and B.

## Results

Soil analysis as summarized in Table 1 points out that both soils A and B are acidic. The organic matter content indicates mineral or organic character of soils A and B, subsequently (Dobrzański and Zawadzki 1995; Fotyma and Mercik 2003). Manganese, cobalt, nickel, copper, zinc, cadmium, and lead content clearly shows that, according to the generally accepted international standards (Council Directive 86/278/EEC; IUSS Working Group WRB 2006), both soils are not contaminated by these metals.

**Fig. 1:**
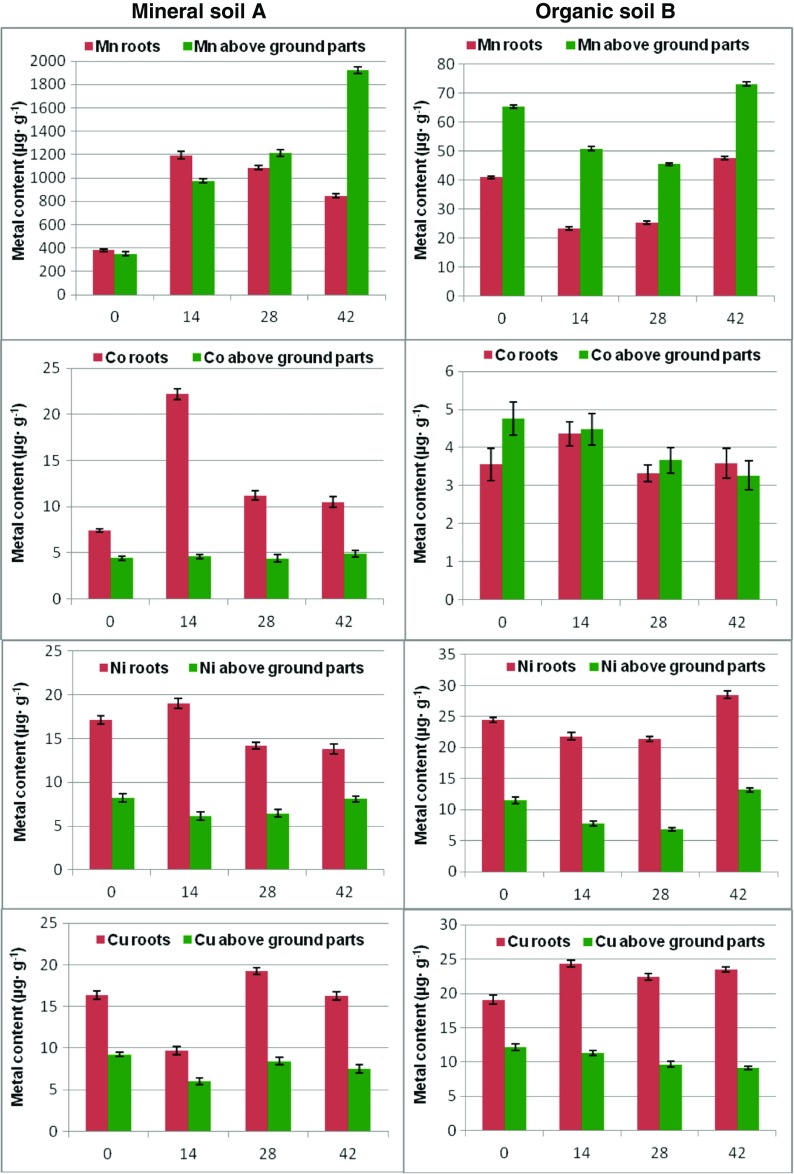

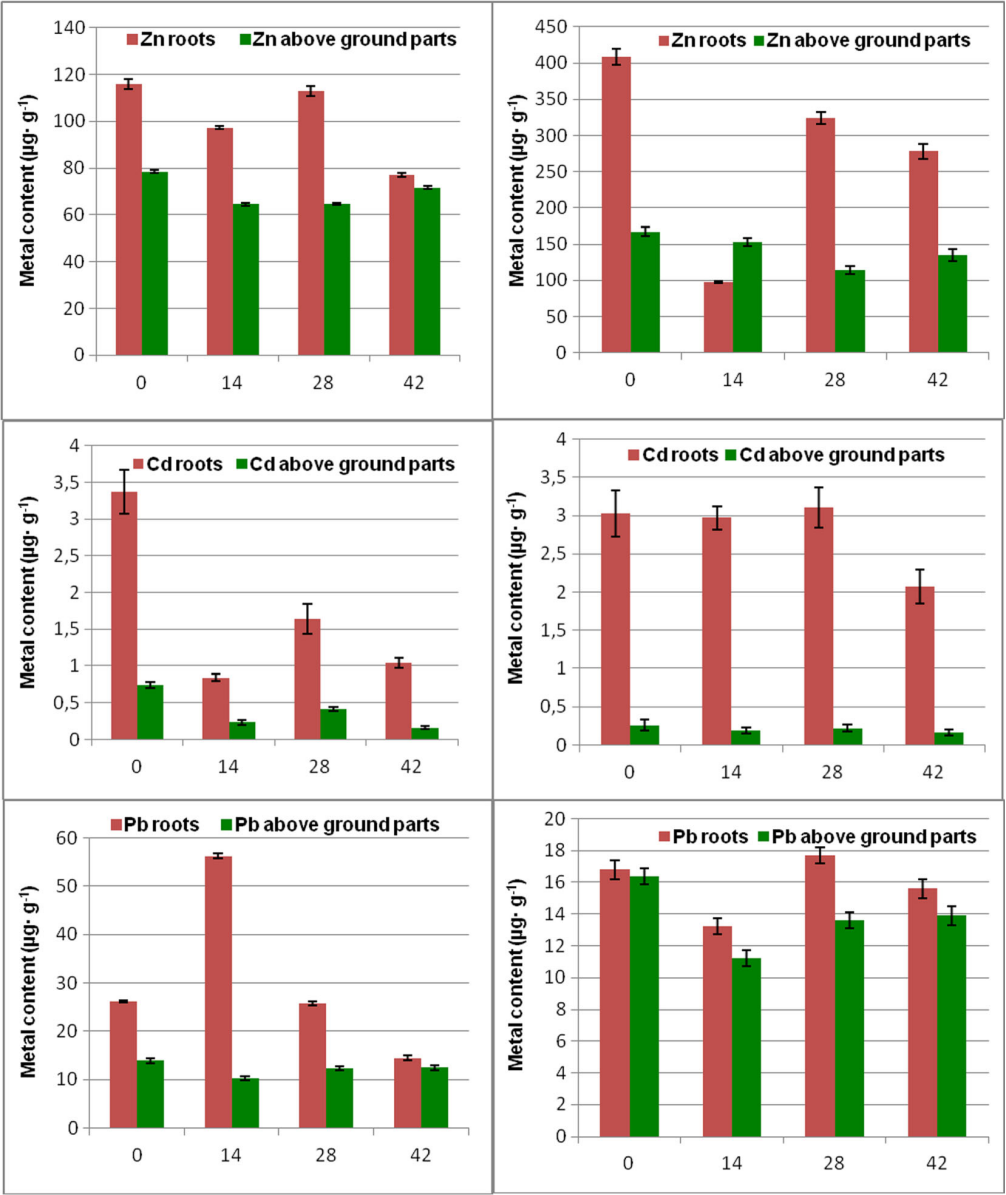
Metal content (μg g^−1^) in roots and aboveground parts of the basil plant displayed against the thiuram contact time (days). Soils A and B are treated separately

Metal content in roots and aboveground parts of the basil plant for soils A and B are summarized in Fig. 1; numerical data are shown in Tables S1 and S2. Analysis of the certified reference material is collected in Table S3. Plants cultivated in the untreated reference mineral soil A accumulate metals mostly in the roots. In organic soil B, manganese and cobalt are concentrated in the aboveground parts, while nickel, copper, zinc, and cadmium accumulate in roots. Our preliminary investigations on *Melissa officinalis* and *Valeriana officinalis* (Adamczyk 2006, 2007; Adamczyk and Jankiewicz 2008) showed that thiuram affects either metal uptake from the soil environment or their further migration within the plant body and prompted us to examine this effect in herbs in a more detailed fashion.

**Figure 2:**
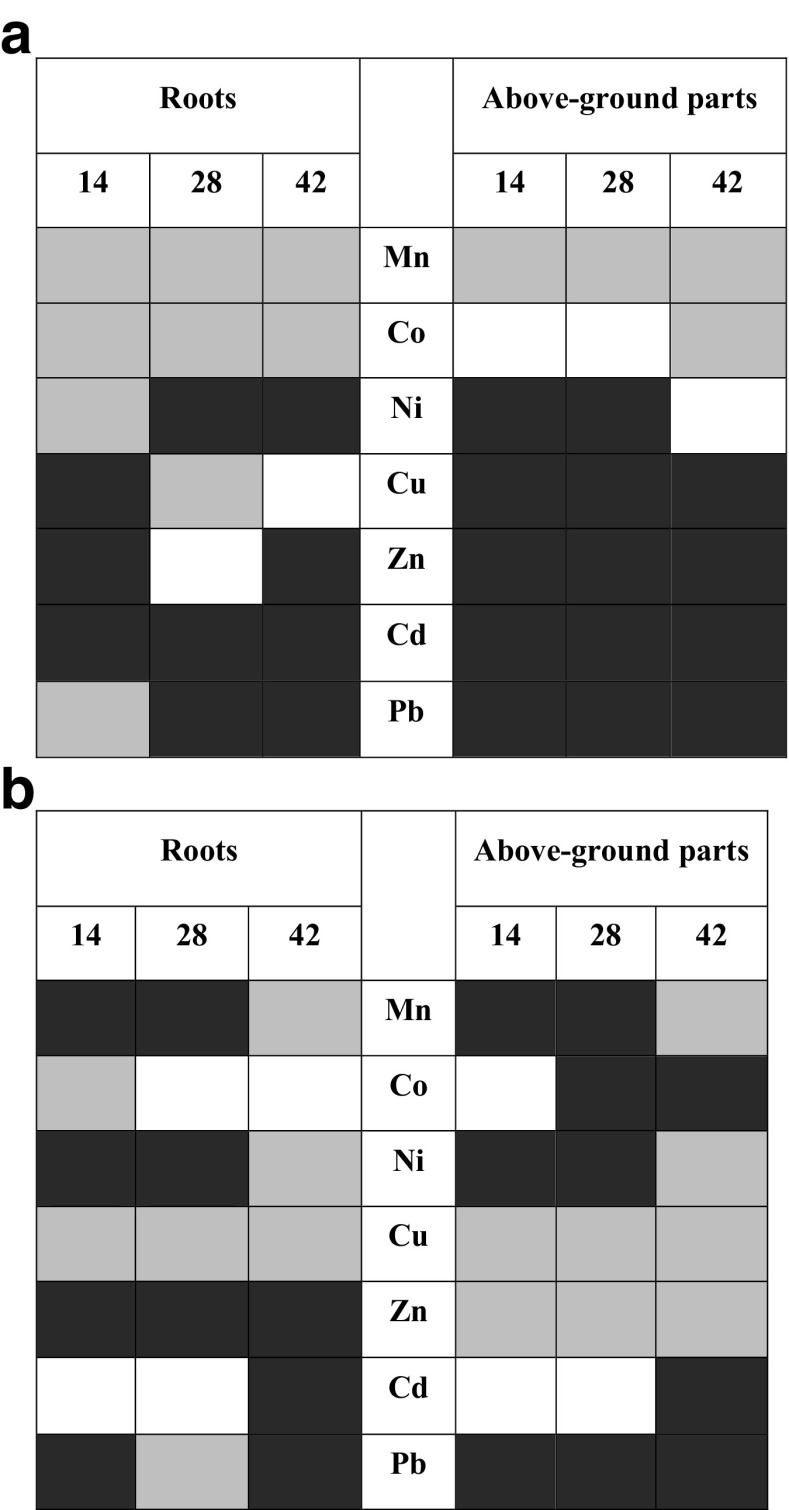
The impact of thiuram contact time (days) on metal content in the basil plant cultivated in mineral soil A (**a**) and organic soil B (**b**) as evaluated by the one-way ANOVA at the 0.95 probability level. Gray color shows combination for which the average metal concentration in a plant tissue increased after the thiuram treatment. Black color represents decrease of respective metal concentration while white indicates no change. Numerical values are given in the supplementary material. Roots and aboveground parts are treated separately

The influence of thiuram contact time on heavy metal accumulation in the plant body was evaluated by ANOVA at the 0.95 probability level (Fig. 2). Detailed numerical data are given in Supplementary material (Tables S4 and S5). The null hypothesis was, whether thiuram treatment influences the metal transfer from soil and their content in roots and aboveground parts of the plant for a particular period of cultivation after the fungicide administration (14, 28, and 42 days). These calculations clearly showed that thiuram affects metal concentration in investigated plants. Major exceptions involved accumulation of cobalt and cadmium at specific times after the addition of thiuram.

**Fig. 3:**
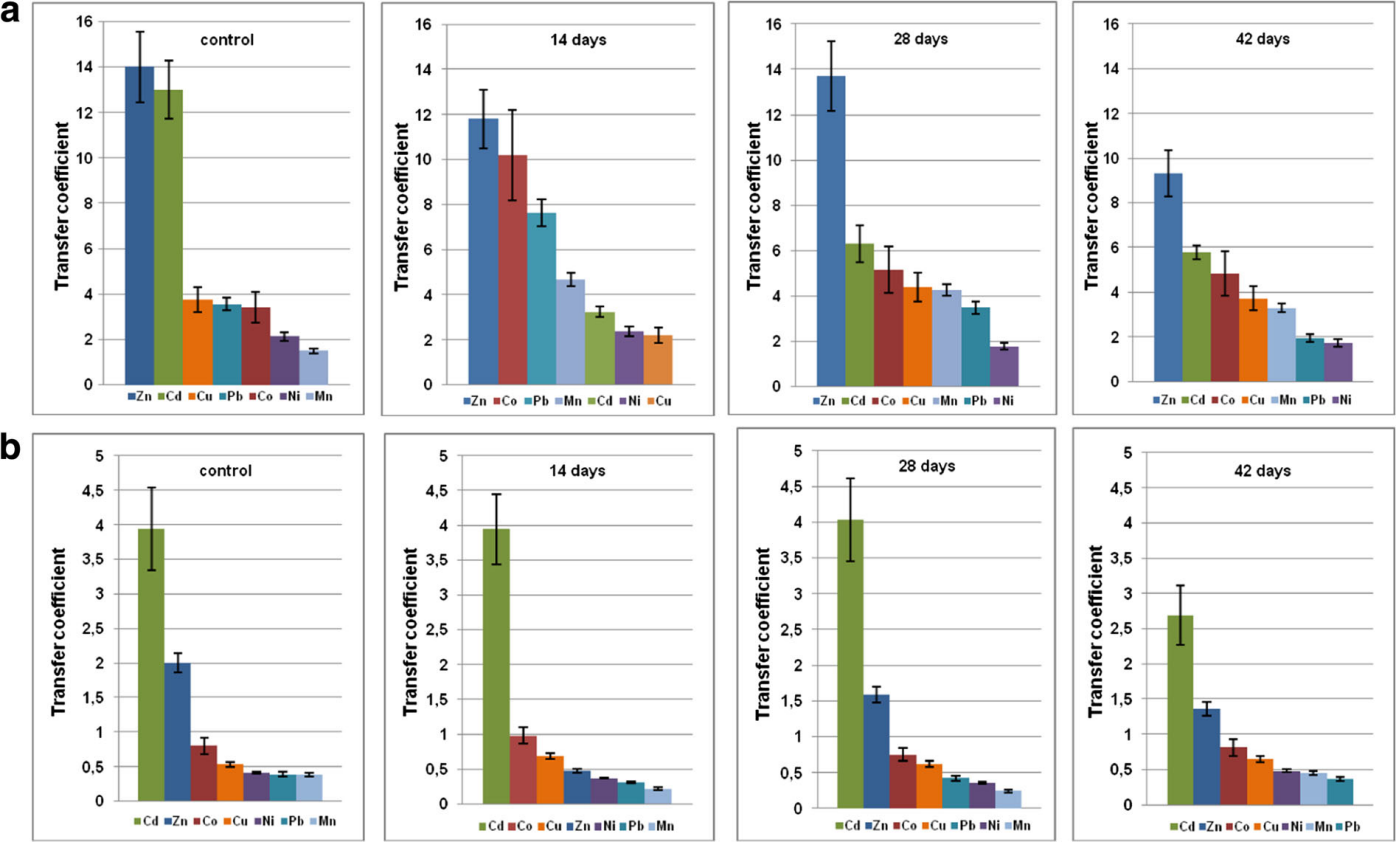
Transfer coefficients (TC) determined for basil plants cultivated in mineral soil A (**a**) and organic soil B (**b**) in the function of time after the fungicide administration. First plot represents untreated control sample

**Fig. 4:**
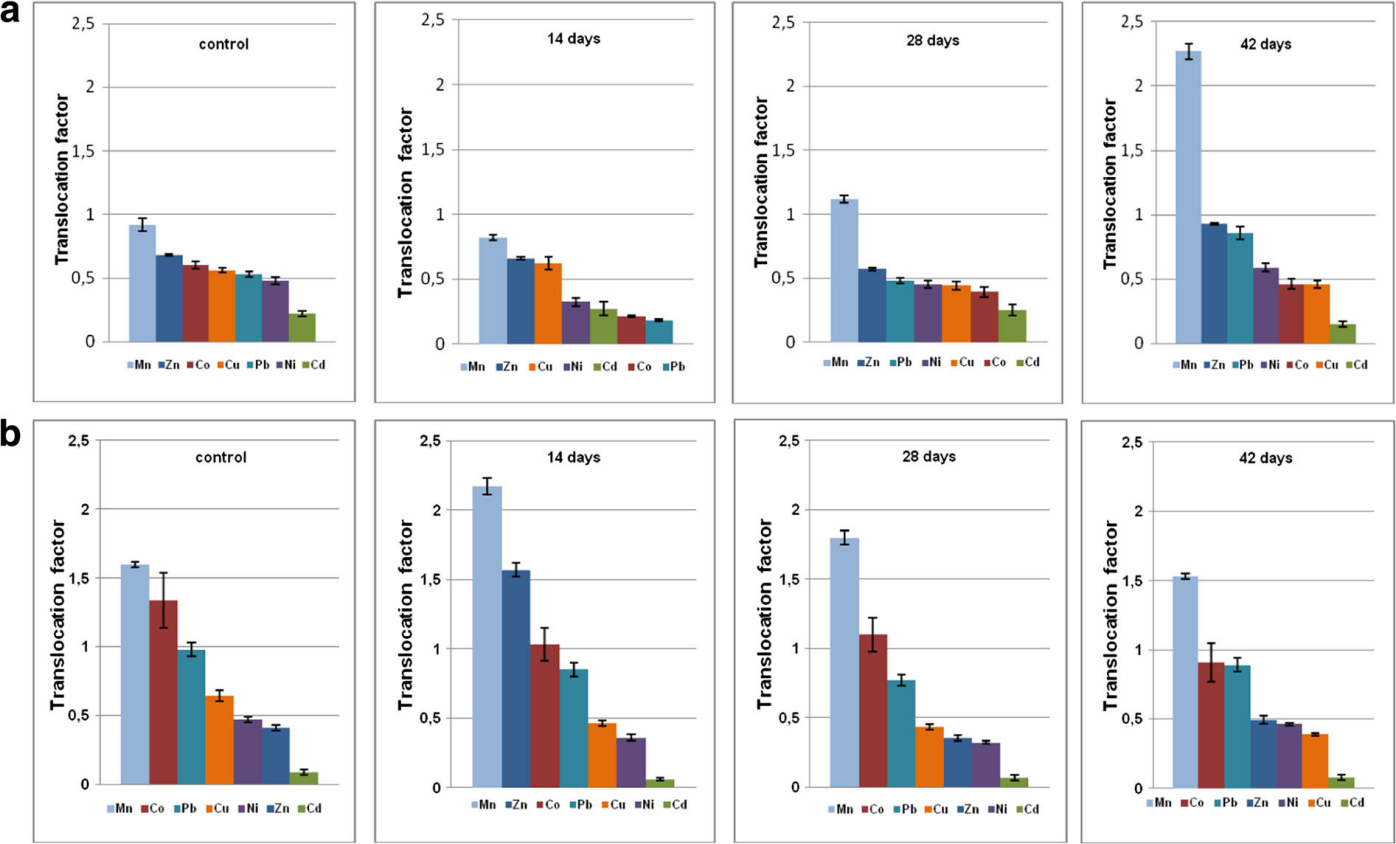
Translocation factor (TF) determined for basil plant cultivated in mineral soil A (**a**) and in organic soil B (**b**) in the function of time after the fungicide administration. First plot represents untreated control sample

Plant uptake of metal from soil was evaluated by its transfer coefficient (TC). This is defined as ratio of particular element concentration in roots to its content in the soil environment (Chen et al. 2016; Galal and Shehata 2015; Liu et al. 2015). Metal distribution inside the plant body was assessed by translocation factor (TF) which is the ratio of element concentration in aboveground part of the plant to that in roots (Shi and Cai 2009; Testiati et al. 2013; Xiao et al. 2015). TCs and TFs computed for four series of cultures in both soils A and B are presented in Figs. 3 and 4, respectively.

**Fig. 5:**
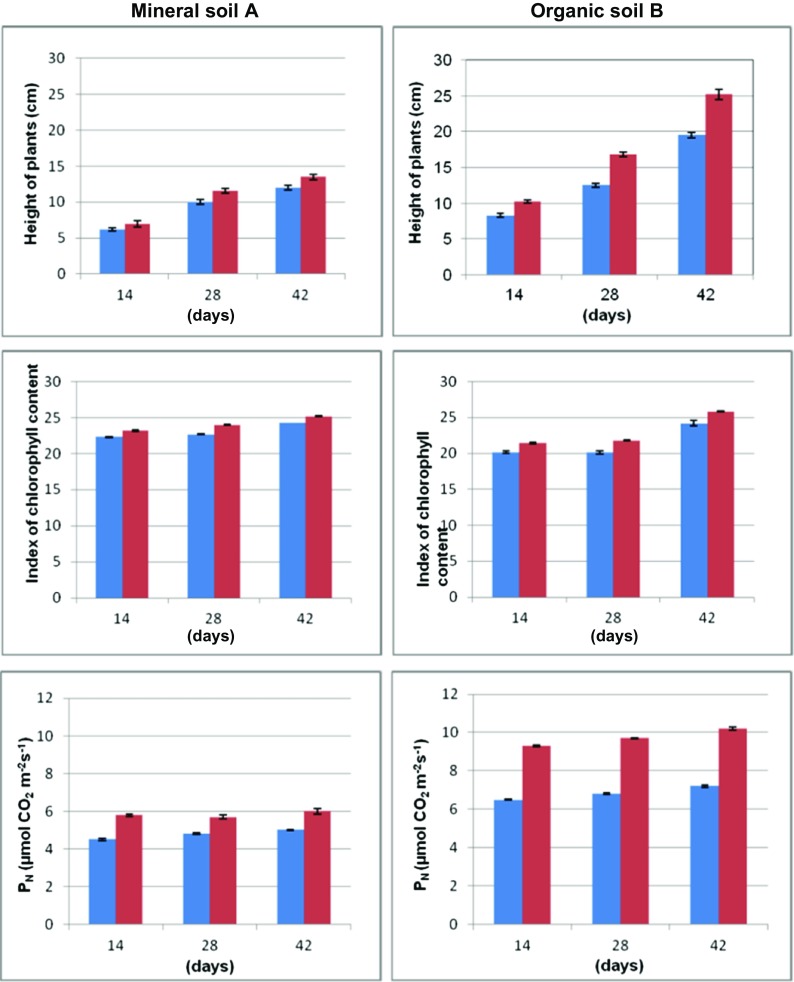

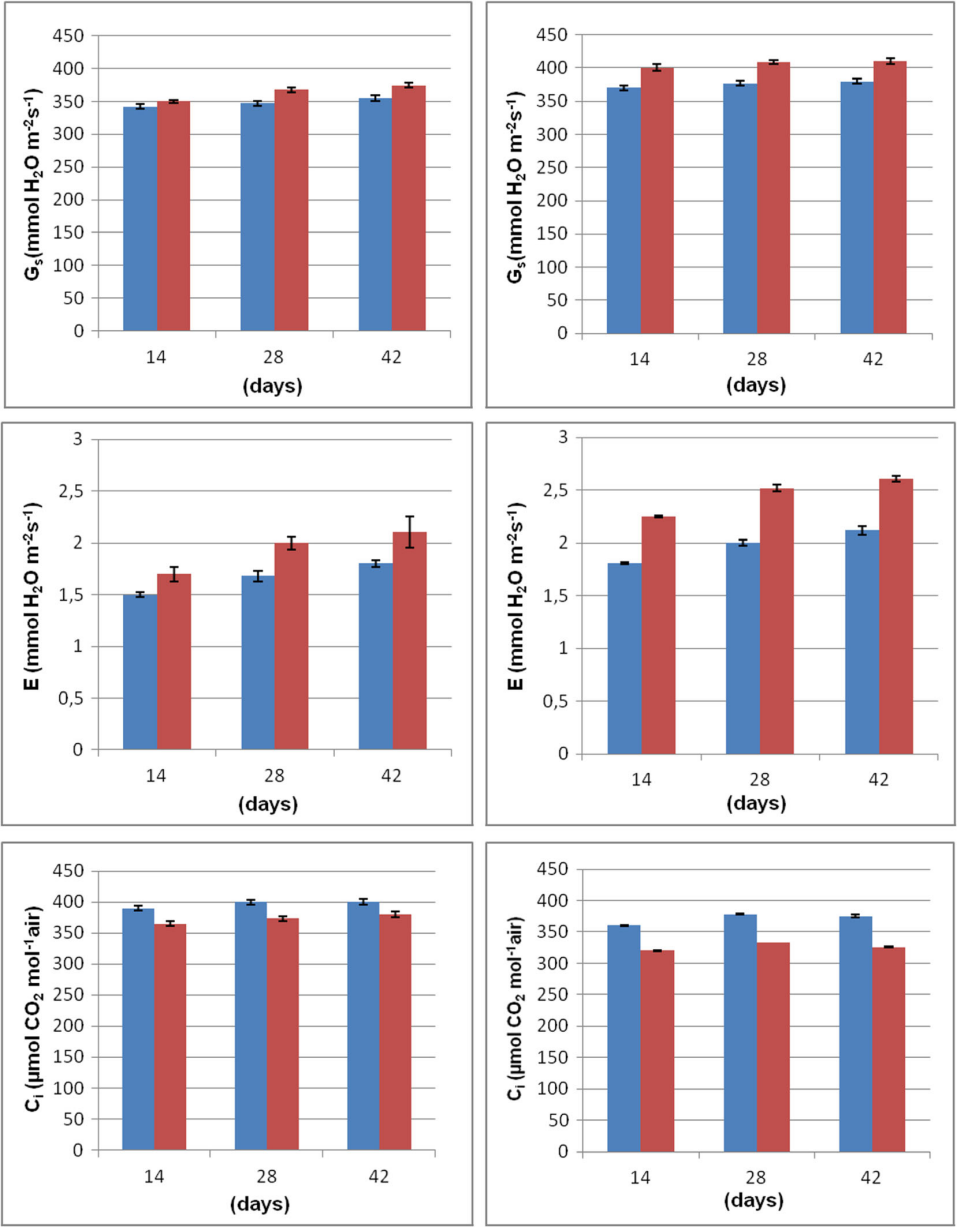
Height of the plant, index of chlorophyll content, net photosynthesis (P_N_), stomatal conductance (G_S_), transpiration (E), and intercellular concentrate CO_2_ (C_i_) calculated for basil grown in mineral and organic soils A and B, respectively. Data for thiuram-treated herbs are given in red. The untreated, control samples are in blue. All parameters were determined repeatedly in 14, 28, and 42 days after the fungicide administration

Metal uptake by plants depends on their health status and should not be discussed without connection to the plant growth. The latter can be conveniently evaluated by the standard photosynthesis indicators, i.e., index of chlorophyll content in leaves, the activity of net photosynthesis, stomatal conductance, transpiration rate and intercellular concentration of CO_2_ (Fig. 5). In this research, all those parameters clearly showed that basil plants were in reasonable growth conditions. However, they are quite sensitive to the type of soil and thiuram contact time. Decreased plant growth rate was observed in mineral soil A while the opposite situation was in the organic soil B. Generally, alterations in the height of basil plants were quite well reflected by photosynthesis indicators. All those parameters were increased after the thiuram administration, especially in organic soil B. As expected, the only exception was intercellular CO_2_ concentration which is decreasing upon the photosynthesis intensification. It means that photosynthesis acceleration is larger than that of stomatal conductance.

## Discussion

It has been documented that fungicide administration influences heavy metal uptake by plants from soil (Adamczyk 2006). However, the impact of thiuram decomposition time has not been widely investigated so far. Thiuram is characterized by limited solubility in water (30 mg L^−1^, 20 °C) and shows a pronounced tendency to adsorb on soil particles; therefore, it is quite safe to groundwater systems. In aquatic conditions at pH = 7, its half-life time is 6 days (Gupta et al. 2012a). Opposite to the model water solutions, in real environment, thiuram degrades more rapidly in acidic soils rich in organic matter. According to Howard (1989), in a humus sandy soil, at pH 3.5, thiuram fully decomposes after 4 to 5 weeks. Rising pH to 7.0 extends that time above 14 weeks (Wauchope et al. 1992; Sharma et al. 2003; Sherif et al. 2011). Thiuram degradation in soil is a complicated process governed by various factors of which moisture, organic content, and microbial activity are of main concern. The major metabolites in soil are dithiocarbamates, dimethylamine, and carbon disulfide (Gupta et al. 2012b).

Thiuram may affect either metal uptake from the soil environment or their further migration within the basil plant. In particular, fungicide administration decreased the content of Zn and Cd during cultivation in mineral soil A, while in organic soil B, the decline was observed for Cd only (Tables S1 and S2). TCs calculated for plants cultivated in the reference, untreated mineral soil A are in the order Zn > Cd > Cu > Pb > Co > Ni > Mn. Thiuram administration interchanges that order for all investigated metals except zinc which is the first in all series, regardless time after the fungicide application. In the untreated reference organic soil B, TCs are in the series Cd > Zn > Co > Cu > Ni > Pb > Mn. Fungicide treatment alters that order for all metal except Cd (Table 2).

**Table 2:** Series of metals ordered according to decreasing transfer coefficients (TCs) and translocation factors (TFs) in the function of thiuram contact time (days). Investigated soils A and B are treated separately.

Thiuram contact time (days)	Mineral soil A	Organic soil B
Transfer coefficients (TCs)		
0	Zn > Cd > Cu > Pb > Co > Ni > Mn	Cd > Zn > Co > Cu > Ni > Pb > Mn
14	Zn > Co > Pb > Mn > Cd > Ni > Cu	Cd > Co > Cu > Zn > Ni > Pb > Mn
28	Zn > Cd > Co > Cu > Mn > Pb > Ni	Cd > Zn > Co > Cu > Pb > Ni > Mn
42	Zn > Cd > Co > Cu > Mn > Pb > Ni	Cd > Zn > Co > Cu > Ni > Mn > Pb
Translocation factors (TFs)		
0	Mn > Zn > Co > Cu > Pb > Ni > Cd	Mn > Co > Pb > Cu > Ni > Zn > Cd
14	Mn > Zn > Cu > Ni > Cd > Co > Pb	Mn > Zn > Co > Pb > Cu > Ni > Cd
28	Mn > Zn > Pb > Ni > Cu > Co > Cd	Mn > Co > Pb > Cu > Zn > Ni > Cd
42	Mn > Zn > Pb > Ni > Cu = Co > Cd	Mn > Co > Pb > Zn > Ni > Cu > Cd

Migration of metals in the plant body may be conveniently examined with the TFs. These factors calculated for basil cultivated in the reference, untreated mineral soil A are in the order Mn > Zn > Co > Cu > Pb > Ni > Cd. For basil grown in organic soil B (without thiuram), TFs are in the series Mn > Co > Pb > Cu > Ni > Zn > Cd. The largest TF decreases were detected for zinc, cobalt, and lead in plants cultivated in mineral soil A 14 days after thiuram administration. The respective TF series was Mn > Zn > Cu > Ni > Cd > Co > Pb (Fig. 4). Plants grown in organic soil B showed TF increase for manganese and zinc 14 days after fungicide administration. The longer contact time (i.e., 28 and 42 days) resulted in TF stabilization. After 42 days, TF values computed for majority of metals were quite close to those reported for untreated soil. Opposite situation was observed in mineral soil A where strong TF increase was identified for manganese, zinc, lead, and nickel after 42-day incubation time. Higher impact of thiuram on heavy metal uptake was found in mineral soil A as compared to organic soil B. This may be related to thiuram persistence in complicated soil matrices. According to Gupta et al. (2012a) high concentration of humic acids (as in soil B) increases the rate of thiuram decay and prompts its lower persistence. Additionally, humic acids shows the well-recognized ability to form stable complexes with metals further reducing mobility and rising their retention in organic soils (Pandey et al. 2000). In organic soil B, thiuram alters heavy metal uptake by the basil plant roots in a diverse way with zinc being the most affected species. Its concentration in roots decreases rapidly in 14 days after fungicide administration. This is associated with zinc migration to the fast-growing green parts of the basil plant. The opposite situation is observed in mineral soil A, where thiuram hampers zinc transport to aboveground parts of herbs and stabilizes its accumulation in roots. In this soil, the highest impact of thiuram is visible in manganese uptake, transport, and accumulation. Its concentration increases in either roots or aboveground parts of basil plant. Interaction of fungicide with rhizosphere microflora often leads to substantial local pH modifications (Mukerji et al. 2006) which are more visible in mineral soil A with buffer capacity lower than that of organic soil B. It is well recognized that in either the soil or the plant cell environment, manganese can exist in a number of chemical forms, namely Mn^+2^ ions and insoluble manganese oxides (Adamczyk-Szabela et al. 2015, Skiba et al. 2017). In acidic conditions, the former are readily available to plants and further prone to migration within the plant body (Adriano 2001; Watmough et al. 2007). Additionally, Mn transfer may also involve superoxide dismutase (SOD) which neutralizes oxygen reactive species (ROS) produced in the plant metabolism. Conserved Mn is an important cofactor which secures the enzyme activity (Whittaker 2010). Metal uptake by roots from soil is strongly dependent on the rhizosphere environment in which bacteria and fungi play the vital role. Additionally, the influence of humic acids cannot be ruled out (Gupta et al. 2016). It is well recognized that mycorrhizal fungi are responsible for nutrients and metal uptake, with zinc and copper being the mostly prone elements (Tinker and Gilden 1983; Habte 2000). Thiuram reduces fungi populations in soil, but its impact on rhizobacteria is complicated and has not been fully understood yet. Obviously, it deserves more attention in the future.

## Conclusions

Heavy metals determined in basil originated from soil environment through the root uptake. They are mobilized in rhizosphere by variety of mechanisms involving roots and microorganism exudates. Obviously, thiuram modifies the mycoflora in the rhizosphere zone and subsequently affects either metal uptake from the soil environment or their further migration within the plant. Notable, those changes are more evident for basil planted in mineral soil A as compared to organic soil B with higher buffering capacity. Additionally, migration of metals may be influenced by the formation of sparingly water-soluble metal-fungicide complexes (Beurskens et al. 1971; Zhao et al. 2003). In particular, the latter are presumable responsible for the high manganese uptake and translocation in plants cultivated on mineral soil A. Thiuram impact on metal migration is highly dependent on its persistence and activity in soil. The highest influence was observed 14 days after fungicide administration. Reduction of thiuram activity and, in consequence, the soil and plant recovery is more visible in organic soil B. This effect may influence heavy metal uptake and their further concentration in plant. It should be taken into the consideration when herbal plantations are to be protected with fungicides.

## Electronic supplementary material

Table S1 (DOC 43 kb)

Table S2 (DOC 42 kb)

Table S3 (DOC 27 kb)

Table S4 (DOC 39 kb)

Table S5 (DOC 38 kb)

## References

[CR1] Adamczyk D (2006). The effect of thiuram on the uptake of lead and copper by *Melissa officinalis*. Environmental Engineering Science.

[CR2] Adamczyk D (2007). The effect of thiuram on the uptake of zinc by *Melissa Officinalis*. Ecological Chemistry and Engineering.

[CR3] Adamczyk D, Jankiewicz B (2008). The effect of thiuram on the uptake of copper, zinc and manganese by *Valeriana officinalis L*. Polish Journal of Environmental Studies.

[CR4] Adamczyk-Szabela D (2015). Influence of thiuram on metal form in soil—determination of copper, zinc and manganese by Tessier’s sequential extraction. JRI: Natural, Medical and Health Sciences.

[CR5] Adamczyk-Szabela D, Markiewicz J, Wolf WM (2015). Heavy metal uptake by herbs. IV. Influence of soil pH on the content of heavy metals in *Valeriana officinalis L. Water*. Air and Soil Pollution.

[CR6] Adigüzel A, Güllüce M, Sengül M, Ögütcü H, Sahin F, Karaman I (2005). Antimicrobial effects of *Ocimum basilicum (Labiatae*) extract. Turkish Journal of Biology.

[CR7] Adriano DC (2001). Trace elements in terrestrial environments.

[CR8] ASTM D2974-00 (2000). Standard test methods for moisture, ash, and organic matter of peat and other organic soils. Method D 2974–00.

[CR9] Basgel S, Erdemoglu SB (2006). Determination of mineral and trace elements in some medicinal herbs and their infusions consumed in Turkey. Science of the Total Environment.

[CR10] Bazaid SA, El-Amoudi MS, Ali EF, Abdel-Hameed ES (2013). Volatile oil studies of some aromatic plants in Taif region. Journal of Medicinal Plants Studies.

[CR11] Beatovic D, Krstic-Miloševic D, Trifunovic S, Šiljegovic J, Glamoclija J, Ristic M, Jelacic S (2015). Chemical composition, antioxidant and antimicrobial activities of the essential oils of twelve *Ocimum basilicum* L. Cultivars Grown in Serbia, Records of Natural Products.

[CR12] Beurskens PT, Cras JA, Noordik JH, Spruijt AM (1971). Crystal and molecular structure of diiodo-N ,N ,N ',N '-tetramethylthiuramdisulphidemercury(II). Journal of Crystal and Molecular Structure.

[CR13] Bianchi JJA, Booij CJH, Tscharntke T (2006). Sustainable pest regulation in agricultural landscapes: a review on landscape composition, biodiversity and natural pest control. Proceeding of the Royal Society B.

[CR14] Bruni R, Pellati F, Bellardi MG, Benvenuti S, Paltrinieri S, Bertaccini A (2005). Herbal drug quality and phytochemical composition of Hypericum perforatum L. affected by ash yellows phytoplasma infection. Journal of Agricultural and Food Chemistry.

[CR15] Bruni R, Bellardi MG, Parrella G (2016). Change in chemical composition of sweet basil (*Ocimum basilicum L.*) essential oil caused by alfalfa mosaic virus. Journal of Phytopathology.

[CR16] Carovic-Stanko K, Orlic S, Politeo O, Strikic F, Kolak I, Milos M, Satovic Z (2010). Composition and antibacterial activities of essentials oils of seven *Ocimum* taxa. Food Chemistry.

[CR17] Chen H, Yuan X, Li T, Hu S, Ji J, Wang C (2016). Characteristics of heavy metal transfer and their influencing factor in different soil-crop systems of the industrialization region, China. Ecotoxicology and Environmental Safety.

[CR18] Council Directive 86/278/EEC (1986). Of 12 June 1986 on the protection of the environment, and in particular of the soil, when sewage sludge is used in agriculture. Official Journal L.

[CR19] Dias MC (2012). Phytotoxicity: an overview of the physiological responses of plants exposed to fungicides. Journal of Botany.

[CR20] Dias MC, Figueiredo P, Duarte IF, Gil AM, Santos C (2014). Different responses of young and expanded lettuce leaves to fungicide Mancozeb: chlorophyll fluorescence, lipid peroxidation, pigments and proline content. Photosynthetica.

[CR21] Dobrzański B, Zawadzki S (1995). Soil Science.

[CR22] Dybczyński R, Danko B, Kulisa K, Maleszewska E, Polkowska-Motrenko H, Samczyński Z, Szopa Z (2004). Preparation and preliminary certification of two new Polish CRMs for inorganic trace analysis. Journal of Radioanalytical and Nuclear Chemistry.

[CR23] Esetlili BÇ, Öztürk B, Çobanoglu Ö, Anaç D (2016). Sweet basil (*Ocimum basilicum L*.) and potassium fertilization. Journal Plant Nutrition.

[CR24] Filipe OMS, Costa CAE, Vidal MM, Santos EBH (2013). Influence of soil copper content on the kinetics of thiram adsorption and on thiram leachability from soils. Chemosphere.

[CR25] Fotyma M, Mercik S (2003). Agricultural chemistry.

[CR26] Galal TM, Shehata HS (2015). Bioaccumulation and translocation of heavy metals by *Plantago major* L. grown in contaminated soils under the effect of traffic pollution. Ecological Indicators.

[CR27] Garcia PC, Rivero RM, Ruiz JM, Romero L (2003). The role of fungicides in the physiology of higher plants: implications for defense responses. Botanical Review.

[CR28] Golcz, A., & Seidler-Łożykowska, K. (2008). Commom basil (*Ocimum basilicum* L.). UP Poznań (in polish).

[CR29] Grayer RJ, Kite GC, Goldstone FJ, Bryan SE, Paton A, Putievsky E (1996). Infraspecific taxonomy and essentials oil chemotypes in sweet basil (*Ocimum basilicum L*.). Phytochemistry.

[CR30] Grzesik M, Romanowska-Duda ZB (2015). Ability of *Cyanobacteria* and microalgae in improvement of metabolic activity and development of willow plants. Polish Journal of Environmental Studies.

[CR31] Gupta B, Rani M, Kumar R (2012). Degradation of thiram in water, soil and plants: a study by high-performance liquid chromatography. Biomedical Chromatography.

[CR32] Gupta B, Rani M, Kumar R, Dureja P (2012). Identification of degradation products of thiram in water, soil and plants using LC-MS technique. Journal of Environ. Science and Health, Part B.

[CR33] Gupta N, Ram H, Kumar B (2016). Mechanism of zinc absorption in plants: uptake, transport, translocation and accumulation. Review in Environmental Science and Biotechnology.

[CR34] Habte M, Silva JA, Uchida R (2000). Mycorrhizal fungi and plant nutrition, from: plant nutrient management in Hawaii’s soils, approaches for tropical and subtropical agriculture. College of Tropical Agriculture and Human Resources.

[CR35] Howard PH (1989). Handbook of environmental fate and exposure data for organic chemicals: pesticides.

[CR36] Hudaib M, Bellardi MG, Rubies-Autonell C, Fiori J, Cavrini V (2001). Chromatographic GC-MS, HPLC and virological evaluations of *Salvia sclarea* infected by BBWVI. Farmaco.

[CR37] Hudaib M, Cavrini V, Bellardi MG, Rubies-Autonell C (2002). Characterization of the essential oils of healthy and virus infected Echinacea Purpurea (L.) Moench plants. *Journal of Essential Oil*. Research.

[CR38] Hussain AI, Anwar F, Hussain Sherazi ST, Przybylski R (2008). Chemical composition, antioxidant and antimicrobial activities of basil (*Ocimum basilicum* L.) essential oils depends on seasonal variations. Food Chemistry.

[CR39] Ismail M (2006). Central properties and chemical composition of *Ocimum basilicum* essential oil. Pharmaceutical Biology.

[CR40] IUSS Working Group WRB (2006). World reference base for soil resources 2006. World Soil Resources Reports No. 103.

[CR41] Janisiewicz WJ, Korsten L (2002). Biological control of postharvest diseases of fruits. Annual Review of Phytopathology.

[CR42] Javanmardi J, Khalighi A, Kashi A, Bais HP, Vivancostefan JM (2002). Chemical characterization of basil (*Ocimum basilicum* L.) found in local accessions and used in traditional medicines in Iran. Journal of Agricultural and Food Chemistry.

[CR43] Kalaji MH, Carpentier R, Allakhverdiev SI, Bosa K (2012). Fluorescence parameters as an early indicator of light stress in barley. Journal of Photochemistry and Photobiology B.

[CR44] Kalaji MH, Schansker G, Ladle RJ, Goltsev V, Bosa K, Allakhverdiev SI, Brestic M, Bussotti F, Calatayud A, Dąbrowski P, Elsheery NI, Ferroni L, Guidi L, Hogewoning SW, Jajoo A, Misra AN, Nebauer SG, Pancaldi S, Penella C, Poli D, Pollastrini M, Romanowska-Duda ZB, Rutkowska B, Serô-Dio J, Suresh K, Szulc W, Tambussi E, Yanniccari M, Zivcak M (2016). Frequently asked questions about chlorophyll fluorescence, the sequel. Photosynthesis Research.

[CR45] Kandil MA, Khatab ME, Ahmed SS, Schnug E (2009). Herbal and essential oil yield of Genovese basil (*Ocimum basilicum L.)* grown with mineral and organic fertilizer sources in Egypt. Journal für Kulturpflanzen.

[CR46] Kitagawa E, Takahashi J, Momose Y, Iwahashi H (2002). Effects of the pesticide thiuram: genome-wide screening of indicator genes by yeast DNA microarray. Environmental Science & Technology.

[CR47] Koba K, Poutouli PW, Raynaud C, Chaumont JP, Sanda K (2009). Chemical composition and antimicrobial properties of different basil essential oils chemotypes from Togo Bangladesh. Journal of Pharmacology.

[CR48] Liu K, Lv J, He W, Zhang H, Cao Y, Dai Y (2015). Major factors influencing cadmium uptake from the soil into wheat plants. Ecotoxicology and Environmental Safety.

[CR49] Malinowska E, Jankowski K (2015). Pesticide residues in some herbs growing in agricultural areas in Poland. Environmental Monitoring and Assessment.

[CR50] Moghaddam AMD, Shayegh J, Mikaili P, Sharaf JD (2011). Antimicrobial activity of essentials oil extract of *Ocimum basilicum* L. leaves on a variety of pathogenic bacteria. Journal of Medicinal Plants Research.

[CR51] Mukerji KG, Manoharachary C, Singh J (2006). Microbial activity in the rhizosphere.

[CR52] Muralidharan A, Dhananjayan R (2004). Cardiac stimulant activity of *Ocimum basilicum* Linn. Extracts. Indian Journal of Pharmacology.

[CR53] Nelson DW, Sommers LE, Page AL (1996). Total carbon, organic carbon, and organic matter. Methods of soil analysis, part 2.

[CR54] Nurzyńska-Wierdak R (2012). *Ocimum basilicum* L.—valuable spice plant, medicinal and essential oil crop. Annales Universitatis Mariae Curie-Skłodowska.

[CR55] Paduraru I, Paduraru O, Miron A (2008). Assessment of antioxidant activity of *Basilici herba* aqueous extract-in vitro study. Farmácia.

[CR56] Pandey AK, Pandey SD, Misra V (2000). Stability constants of metal-humic acid complexes and its role in environmental detoxification. Ecotoxicology and Environmental Safety.

[CR57] Petit AN, Fontaine F, Clément C, Vaillant-Gaveau N (2008). Photosynthesis limitations of grapevine after treatment with the fungicide fludioxonil. Journal of Agricultural and Food Chemistry.

[CR58] Piotrowski K, Romanowska-Duda ZB, Grzesik M (2016). How Biojodis and cyanobacteria alleviate the negative influence of predicted environmental constraints on growth and physiological activity of corn plants. Polish Journal of Environmental Studies.

[CR59] PN-ISO 10381-4:2007 Soil quality - sampling - part 4: rules for procedure during the research areas of natural, semi-natural and cultivated.

[CR60] PN-ISO 10390:1997 Agricultural chemical analysis of the soil. Determination of pH.

[CR61] PN-ISO 11259:2001 Soil quality—a simplified description of the soil.

[CR62] Politeo O, Jukic M, Milos M (2007). Chemical composition and antioxidant capacity of free volatile aglycones from basil (*Ocimum basilicum* L.) compared with its essential oil. Food Chemistry.

[CR63] Salles Trevisan MT, Vasconelos Silva MG, Pfundstein B, Spiegelhalder B, Owen RW (2006). Characterization of the volatile pattern and antioxidant capacity of essential oils from different species of the genus *Ocimum*. Journal of Agricultural and Food Chemistry.

[CR64] Sau-Man Po E, Ho JW (1997). Effect of pyrrolidine dithiocarbamate on photo-induced proton transport through chloroplast membranes. Biochemistry and Molecular Biology International.

[CR65] Schumacher BA (2002). Methods for the determination of total organic carbon (TOC) in soils and sediments.

[CR66] Sekar K, Thangaraj S, Saravana Babu S, Harisaranraj R, Suresh K (2009). Phytochemical constituent and antioxidant activity of extract from the leaves of *Ocimum basilicum*. Journal of Phytology.

[CR67] Sexton AC, Howlett BJ (2006). Parallels in fungal pathogenesis on plant and animal hosts. Eukaryotic Cell.

[CR68] Sharma VK, Aulakh JS, Malik AK (2003). Thiram: Degradation, applications and analytical methods. Journal of Environmental Monitoring.

[CR69] Sherif AM, Elhussein AA, Osman AG (2011). Biodegradation of fungicide thiram (TMTD) in soil under laboratory conditions. American Journal of Biotechnology and Molecular Sciences.

[CR70] Shi GR, Cai QS (2009). Photosynthetic and anatomic responses of peanut leaves to zinc stress. Biologia Plantarum.

[CR71] Skiba E, Kobyłecka J, Wolf WM (2017). Influence of 2,4-D and MCPA herbicides on uptake and translocation of heavy metals in wheat (*Triticum aestivum* L.). Environmental Pollution.

[CR72] Snelders E, Melchers WJ, Verweij PE (2011). Azole resistance in Aspergillus fumigatus: a new challenge in the management of invasive aspergillosis?. Future Microbiology.

[CR73] Stefan M, Zamfirache MM, Padurariu C, Trută E, Gostin I (2013). The composition and antibacterial activity of essential oils in three Ocimum species growing in Romania. Central European Journal of Biology.

[CR74] Taie HAA, Salama ZAR, Radwan S (2010). Potential activity of basil plants as a source of antioxidants and anticancer agents as affected by organic and bio-organic fertilization. Notulae Botanicae Horti Agrobotanici Cluj-Napoca.

[CR75] Testiati E, Parinet J, Massiani C, Laffont-Schwob I, Rabier J, Pfeifer HR, Lenoble V, Prudent P (2013). Trace metal and metalloid contamination levels in soils and two native plant species of a former industrial site: Evaluation of thephytostabilization potential. Journal of Hazardous Materials.

[CR76] Tinker PB, Gilden A, Robb DA, Pierpoint WS (1983). Mycorrhizal fungi and ion uptake. Metals and micronutrients, uptake and utilization by plants.

[CR77] Tsai KD, Lin BR, Perng DS, Wei JC, Yu YW, Cherng JM (2011). Immunomodulatory effects of aqueous extract of *Ocimum basilicum* (Linn.) and some of its constituents on human immune cells. Journal of Medicinal Plants Research.

[CR78] Victoriano LI (2000). The reactivity of metal species towards thiuram sulfides: an alternative route to the syntheses of metal dithiocarbamates. Coordination Chemistry Reviews.

[CR79] Victoriano LI (2000). The reaction of copper and iron species with thiuram sulfides: copper and iron dithiocarbamate derivatives. Polyhedron.

[CR80] Watmough SA, Eimers MC, Dillon PJ (2007). Manganese cycling in central Ontario forests: Response to soil acidification. Applied Geochemistry.

[CR81] Wauchope RD, Buttler TM, Hornsby AG, Augustijn-Beckers PWM, Burt JP (1992). The SCS/ARS/CES pesticide properties database for environmental decision-making. Reviews of Environmental Contamination and Toxicology.

[CR82] Whittaker JW (2010). Metal uptake by manganese dismutase. Biochimica et Biophysica Acta.

[CR83] Xiao JX, Huang YY, Wang L, Huang LF, Yu YL, Zhou YH, Yu JQ (2006). Pesticides induced depression of photosynthesis was alleviated by 24-epibrassinolide pretreatment in *Cucumis sativus* L. Pesticide Biochemistry and Physiology.

[CR84] Xiao R, Bai J, Lu Q, Zhao Q, Gao Z, Wen X, Liu X (2015). Fractionation, transfer and ecological risks of heavy metals in riparian and ditch wetlands across a 100-year chronsequence of reclamation in estuary of China. Science of the Total Environment.

[CR85] Zechini D’Aulerio A, Zambonelli A, Bianchi A, Albasini A (1995). Micromorphological and chemical investigation into the effects of fungal diseases on *Melissa officinalis L*., *Menthapiperita L.* and *Salvia officinalis L*. Journal of Phytopathology.

[CR86] Zeggwagh NA, Sulpice T, Eddouks M (2007). Anti-hyperglycaemic and hypolipidemic effects of *Ocimum basilicum* aqueous extract in diabetic rats. American Journal of Pharmacology and Toxicology.

[CR87] Zhao YG, Zheng XW, Huang ZY, Yang MM (2003). Voltammetric study on the complex of thiram-copper(II) and its application. Analytica Chimica Acta.

