# Supplementary material for: Heavy Metal Uptake by Herbs. V. Metal Accumulation and Physiological Effects Induced by Thiuram in Ocimum basilicum L
Source: Water Air Soil Pollut. 2017 Aug 17;228(9):334. doi: 10.1007/s11270-017-3508-0 (PMC5561165; doi:10.1007/s11270-017-3508-0)
Supplement: Supplementary file 1 — (DOC 43 kb) [file 11270_2017_3508_MOESM1_ESM.doc]

Table S1. Metals content in roots and above-ground parts of the basil plants cultivated in the either raw or treated with thiuram mineral soil A (mean ± SE, n=5).

| Metal | Part of the plant | Metals content in plants (µg·g^-1^) | | | |
| --- | --- | --- | --- | --- | --- |
|  |  | Raw soil | Soil treated with thiuram | | |
|  |  |  | 2 weeks | 4 weeks | 6 weeks |
| **Mn** | Above-ground | 350±19 | 975±15 | 1214±26 | 1921±29 |
|  | Roots | 381±10 | 1194±31 | 1088±18 | 846±18 |
| **Co** | Above-ground | 4.41±0.22 | 4.60±0.20 | 4.40±0.41 | 4.89±0.33 |
|  | Roots | 7.41±0.16 | 22.2±0.6 | 11.2±0.5 | 10.5±0.6 |
| **Ni** | Above-ground | 8.25±0.46 | 6.13±0.44 | 6.45±0.44 | 8.12±0.33 |
|  | Roots | 17.1±0.5 | 19.0±0.6 | 14.2±0.4 | 13.8±0.6 |
| **Cu** | Above-ground | 9.25±0.28 | 6.00±0.37 | 8.44±0.48 | 7.48±0.50 |
|  | Roots | 16.4±0.5 | 9.70±0.53 | 19.2±0.4 | 16.3±0.5 |
| **Zn** | Above-ground | 78.5±0.6 | 64.5 ±0.7 | 64.7±0.6 | 71.6±0.6 |
|  | Roots | 116±2 | 97.3±0.7 | 113±2 | 77.2±0.8 |
| **Cd** | Above-ground | 0.74±0.04 | 0.23±0.04 | 0.41±0.03 | 0.16±0.02 |
|  | Roots | 3.37±0.3 | 0.84±0.05 | 1.64±0.2 | 1.04±0.07 |
| **Pb** | Above-ground | 13.9±0.5 | 10.3±0.4 | 12.3±0.4 | 12.5±0.5 |
|  | Roots | 26.2±0.3 | 56.3±0.5 | 25.7±0.4 | 14.5±0.5 |
