# Supplementary material for: Heavy Metal Uptake by Herbs. V. Metal Accumulation and Physiological Effects Induced by Thiuram in Ocimum basilicum L
Source: Water Air Soil Pollut. 2017 Aug 17;228(9):334. doi: 10.1007/s11270-017-3508-0 (PMC5561165; doi:10.1007/s11270-017-3508-0)
Supplement: Supplementary file 2 — (DOC 42 kb) [file 11270_2017_3508_MOESM2_ESM.doc]

Table S2. Metals content in roots and above-ground parts of the basil plants cultivated in the either raw or treated with thiuram organic soil B (mean ± SE, n=5).

| **Metal** | Part of the plant | Metals content in plants (µg·g^-1^) | | | |
| --- | --- | --- | --- | --- | --- |
|  |  | Raw soil | Soil treated with thiuram | | |
|  |  |  | 2 weeks | 4 weeks | 6 weeks |
| **Mn** | Above-ground | 65.4±0.5 | 50.8±0.7 | 45.5±0.4 | 73.2±0.6 |
|  | Roots | 41.0±0.4 | 23.4±0.5 | 25.3±0.6 | 47.7±0.5 |
| **Co** | Above-ground | 4.76±0.43 | 4.48±0.42 | 3.67±0.34 | 3.27±0.38 |
|  | Roots | 3.56±0.42 | 4.36±0.32 | 3.33±0.22 | 3.58±0.39 |
| **Ni** | Above-ground | 11.5±0.5 | 7.79±0.35 | 6.84±0.32 | 13.2±0.3 |
|  | Roots | 24.5±0.4 | 21.8±0.6 | 21.4±0.4 | 28.5±0.6 |
| **Cu** | Above-ground | 12.2±0.5 | 11.3±0.4 | 9.65±0.43 | 9.13±0.26 |
|  | Roots | 19.1±0.7 | 24.4±0.5 | 22.4±0.5 | 23.5±0.4 |
| **Zn** | Above-ground | 167±6 | 153±5 | 114±5 | 135±8 |
|  | Roots | 408±11 | 97.4±1.2 | 324±8 | 278±10 |
| **Cd** | Above-ground | 0.26±0.07 | 0.19±0.04 | 0.22±0.05 | 0.16±0.04 |
|  | Roots | 3.03±0.30 | 2.97±0.15 | 3.10±0.26 | 2.07±0.22 |
| **Pb** | Above-ground | 16.4±0.5 | 11.2±0.5 | 13.6±0.5 | 13.9±0.6 |
|  | Roots | 16.8±0.6 | 13.2±0.5 | 17.7±0.5 | 15.6±0.6 |
