# Supplementary material for: Heavy Metal Uptake by Herbs. V. Metal Accumulation and Physiological Effects Induced by Thiuram in Ocimum basilicum L
Source: Water Air Soil Pollut. 2017 Aug 17;228(9):334. doi: 10.1007/s11270-017-3508-0 (PMC5561165; doi:10.1007/s11270-017-3508-0)
Supplement: Supplementary file 3 — (DOC 27 kb) [file 11270_2017_3508_MOESM3_ESM.doc]

Table S3. Metals content in the certified reference material ($\bar{\text{x}}\pm\text{ts}_{\bar{\text{x}}}$; p = 0.95, n = 6).

| **Metal** | **Certified value**  **µg/g** | **Found**  **µg/g** | **Recovery**  **%** |
| --- | --- | --- | --- |
| **Manganese** | 191 ± 12 | 179 ± 9 | 94 |
|  |  |  |  |
| **Cobalt** | 0.210 ± 0.025 | 0.194 ± 0.011 | 92 |
| **Nickel** | 1.57 ±0.16 | 1.46 ± 0.22 | 93 |
| **Copper** | 7.77 ± 0.53 | 7.38 ± 0.38 | 95 |
| **Zinc** | 33.5 ± 2.1 | 34.5 ± 0.9 | 103 |
| **Cadmium** | 0.199 ± 0.015 | 0.209 ± 0.009 | 105 |
| **Lead** | 2.16 ± 0.23 | 2.04 ± 0.17 | 94 |
