# Supplementary material for: Heavy Metal Uptake by Herbs. V. Metal Accumulation and Physiological Effects Induced by Thiuram in Ocimum basilicum L
Source: Water Air Soil Pollut. 2017 Aug 17;228(9):334. doi: 10.1007/s11270-017-3508-0 (PMC5561165; doi:10.1007/s11270-017-3508-0)
Supplement: Supplementary file 4 — (DOC 39 kb) [file 11270_2017_3508_MOESM4_ESM.doc]

Table S4. The impact of thiuram contact time (days) on metal content in the basil plant cultivated in soil A as evaluated by the one-way ANOVA.

| **Roots** | | |  | **Above-ground parts** | | |
| --- | --- | --- | --- | --- | --- | --- |
| **14** | **28** | **42** |  | **14** | **28** | **42** |
| F=3041,446  p=1,3·10^-11^ | F=5754,767  p=1,02·10^-12^ | F=2576,915  p=2,51·10^-11^ | **Mn** | F=3408,40  p=8,23·10^-12^ | F=3624,91  p=6,44·10^-12^ | F=10575,26  p=8,93·10^-14^ |
| F=3253,409  p=9,91·10^-12^ | F=252,3746  p=2,47·10^-7^ | F=93,63333  p=1,08·10^-5^ | **Co** | F=2,0682  p=1,88·10^-1^ | F=0,003347  p=9,55·10^-1^ | F=7,4543  p=2,58·10^-2^ |
| F=28,503  p=6,95·10^-4^ | F=100,311  p=8,39·10^-6^ | F=85,527  p=1,52·10^-5^ | **Ni** | F=54,869  p=7,57·10^-5^ | F=39,92016  p=2,28·10^-4^ | F=0,2790  p=6,11·10^-1^ |
| F=456,744  p=2,42·10^-8^ | F=99,850  p=8,54·10^-6^ | F=0,0693  p=7,99·10^-1^ | **Cu** | F=235,463  p=3,23·10^-7^ | F=9,9510  p=1,35·10^-2^ | F=45,791  p=1,43·10^-4^ |
| F=278,990  p=1,67·10^-7^ | F=4,4521  p=6,78·10^-2^ | F=1178,785  p=5,66·10^-10^ | **Zn** | F=628,047  p=6,9·10^-9^ | F=1330,48  p=3,5·10^-10^ | F=322,853  p=9,44·10^-8^ |
| F=300,507  p=1,25·10^-7^ | F=102,459  p=7,75·10^-6^ | F=249,253  p=2,59·10^-7^ | **Cd** | F=299,547  p=1,27·10^-7^ | F=168,1  p=1,19·10^-6^ | F=699,843  p=4,48·10^-9^ |
| F=15161,13  p= 2,12·10^-14^ | F=5,9559  p=4,05·10^-2^ | F=2281,134  p=4,08·10^-11^ | **Pb** | F=136,3639  p= 2,64·10^-6^ | F=31,79196  p=4,88·10^-4^ | F=18,38569  p=2,66·10^-3^ |
