# Supplementary material for: Heavy Metal Uptake by Herbs. V. Metal Accumulation and Physiological Effects Induced by Thiuram in Ocimum basilicum L
Source: Water Air Soil Pollut. 2017 Aug 17;228(9):334. doi: 10.1007/s11270-017-3508-0 (PMC5561165; doi:10.1007/s11270-017-3508-0)
Supplement: Supplementary file 5 — (DOC 38 kb) [file 11270_2017_3508_MOESM5_ESM.doc]

**Table S5. The impact of thiuram contact time (days) on metal content in the basil plant cultivated in soil B as evaluated by the one-way ANOVA.**

| **Roots** | | |  | **Above-ground parts** | | |
| --- | --- | --- | --- | --- | --- | --- |
| **14** | **28** | **42** |  | **14** | **28** | **42** |
| F=2711,962  p=2,05·10^-11^ | F=1942,343  p=7,75·10^-11^ | F=411,081  p=3,66·10^-8^ | **Mn** | F=1600,300  p=1,68·10^-10^ | F=4283,266  p=3,31·10^-12^ | F=483,126  p=1,94·10^-8^ |
| F=11,5131  p=9,46·10^-3^ | F=1,1378  p=3,17·10^-1^ | F=0,0087  p=9,28·10^-1^ | **Co** | F=1,0935  p=3,26·10^-1^ | F=19,7577  p=2,15·10^-3^ | F=34,0717  p=3,88·10^-4^ |
| F=59,4588  p=5,69·10^-5^ | F=118,350  p=4,51·10^-6^ | F=158,1028  p=1,5·10^-6^ | **Ni** | F=206,050  p=5,41·10^-7^ | F=353,207  p=6,64·10^-7^ | F=47,6038  p=1,25·10^-4^ |
| F=224,028  p=3,92·10^-7^ | F=85,6132  p=1,51·10^-5^ | F=154,495  p=1,64·10^-6^ | **Cu** | F=10,2160  p=1,27·10^-2^ | F=74,3023  p=2,54·10^-5^ | F=145,208  p=2,08·10^-6^ |
| F=4422,859  p=2,91·10^-12^ | F=247,579  p=2,66·10^-7^ | F=374,871  p=5,26·10^-8^ | **Zn** | F=16,7053  p=3,50·10^-3^ | F=231,7657  p=3,44·10^-7^ | F=52,1169  p=9,07·10^-5^ |
| F=0,1677  p=6,93·10^-1^ | F=0,0245  p=8,80·10^-1^ | F=33,0287  p=4,30·10^-4^ | **Cd** | F=4,2160  p=7,41·10^-2^ | F=1,2520  p=2,96·10^-1^ | F=8,3457  p=2,02·10^-2^ |
| F=100,246  p= 8,41·10^-6^ | F=7,3376  p=2,67·10^-2^ | F=9,2164  p=1,62·10^-2^ | **Pb** | F=305,0255  p= 1,18·10^-7^ | F=86,27352  p=1,47·10^-5^ | F=60,0728  p=5,48·10^-5^ |
